# The Dual Nature of Super Women: The Narratives of African American Dementia Caregivers

**DOI:** 10.1093/geroni/igaf122.2718

**Published:** 2025-12-31

**Authors:** Shanae Rhodes, Pamela Recto, Janna Lesser

**Affiliations:** The University of Texas Health Science Center at San Antonio, San Antonio, Texas, United States; UT Health San Antonio, San Antonio, Texas, United States; UT Health San Antonio, San Antonio, Texas, United States

## Abstract

African Americans are twice as likely to be affected by dementia, with most caregivers being women. However, African American women remain underrepresented in dementia research. Literature shows that while African American caregivers report worse physical health, they tend to fare better psychologically regarding caregiver burden compared to other racial groups. Despite this, inconsistencies exist regarding their mental health outcomes. Some studies suggest that African American caregivers use culturally derived coping mechanisms, such as the “superwoman” role, to suppress negative emotions. This coping strategy, driven by societal expectations and cultural norms, can provide short-term relief but contributes to long-term emotional distress and strain. This paradox underscores the need for a deeper understanding of how cultural factors influence stress and coping in caregiving contexts. The purpose of this mixed-methods study was to explore the experiences of African American female caregivers. This presentation focuses on the qualitative segment, using a descriptive approach with ethnographic strategies. Fifteen individual interviews were conducted via Zoom, generating 1,012 minutes of transcribed data. The analysis, guided by Woods-Giscombe’s Superwoman Schema (SWS), identified categories aligned with each SWS domain. Additionally, a new perceived benefit, “overcoming resentment & trauma,” emerged, potentially adding a new domain to the SWS. These findings provide valuable insights into the challenges and strengths of African American women caregivers, offering a foundation for culturally sensitive interventions to support this underserved group.

